# Diagnosis of Brugada syndrome affects quality of life and psychological status

**DOI:** 10.3389/fcvm.2024.1429814

**Published:** 2024-07-03

**Authors:** Paola Berne, Francesca Usai, Etelvino Silva, Irene Melis, Tatiana Fancello, Alessandra Onida, Pierluigi Merella, Francesco Figus, Josep Brugada, Gavino Casu

**Affiliations:** ^1^ Cardiology Department, Ospedale Santissima Annunziata, Azienda Ospedaliera Universitaria, Sassari, Italy; ^2^ Cardiology Department, Hospital Universitario Puerta del Mar, Cadiz, Spain; ^3^ Instituto de Investigación e Innovación Biomédica de Cádiz, Grupo GADICOR, Hospital Universitario Puerta del Mar, Universidad de Cádiz, Cadiz, Spain; ^4^ Integrated Assistance and Territorial Evaluation Unit, ASL1, Sassari, Italy; ^5^ Neurology Department, Ospedale San Francesco, ASL3, Nuoro, Italy; ^6^ Cardiovascular Institute, Hospital Clínic, Barcelona, Spain; ^7^ Pediatric Arrhythmia Unit, Hospital Sant Joan de Déu, Barcelona, Spain; ^8^ Department of Medicine, University of Barcelona, Barcelona, Spain; ^9^ Medical School, Department of Cardiovascular Diseases, University of Sassari, Sassari, Italy

**Keywords:** Brugada syndrome, sudden cardiac death, quality of life, psychological status, anxiety, resilience, perceived stress

## Abstract

**Background:**

Chronic diseases have a negative impact on quality of life (QOL) and psychological health. There are limited related data regarding this topic in Brugada syndrome (BrS). We evaluated the effects of the diagnosis of BrS on health-related QOL and psychological status among patients and their relatives.

**Methods:**

Patients with BrS and their relatives underwent psychological evaluation at diagnosis (T0), 1 and 2 years after diagnosis (T1 and T2) using questionnaires on mental QOL, anxiety, depression, stress, post-traumatic stress, and resilience resources.

**Results:**

Sixty-one patients and 39 relatives were enrolled. Compared with controls, patients showed increased physical QOL (54.1 ± 6.5 vs. 50.1 ± 8.0, *p* = 0.014), reduced mental QOL (43.2 ± 11.8 vs. 49.6 ± 9.1, *p* = 0.018) and increased anxiety (9.9 ± 6.6 vs. 6.9 ± 7.7, *p* = 0.024) at T0; reduced resilience scores (3.69 ± 0.40 vs. 3.96 ± 0.55, *p* = 0.008) at T1; and reduced resilience (3.69 ± 0.35 vs. 3.96 ± 0.55, *p* = 0.019) and increased anxiety scores (16.4 ± 12.8 vs. 6.9 ± 7.7, *p* = 0.006) at T2. Relatives presented higher stress (17.63 ± 3.77 vs. 12.90 ± 6.0, *p* = 0.02) at T0 and higher anxiety scores at T0 (13.5 ± 7.6 vs. 6.9 ± 7.7, *p* < 0.001), T1 (12.0 ± 8.7 vs. 6.9 ± 7.7, *p* = 0.005), and T2 (16.4 ± 12.8 vs. 6.9 ± 7.7, *p* = 0.006) than controls. Female sex was significantly independently associated with worse mental QOL scores in patients at T0 (odds ratio = 0.10; 95% confidence interval = 0.05–0.94; *p* = 0.04).

**Conclusions:**

The diagnosis of BrS impairs the QOL and psychological status of patients and their relatives. Female sex is independently associated with worse mental QOL in patients at diagnosis.

## Introduction

Brugada syndrome (BrS) is an inherited condition characterized by coved-type ST segment elevation followed by negative T waves in the right precordial leads V1 and/or V2 in the absence of overt structural heart disease. Patients with BrS have increased risk of sudden cardiac death (SCD) secondary to sustained ventricular arrhythmias, most of these events occur at the fourth decade of life in otherwise healthy individuals (1). Mutations in the *SCN5A* gene, encoding the *α*-subunit of the Nav1.5 sodium channel are found in circa 20% of patients. After the diagnosis of BrS, patients must avoid certain drugs and promptly treat fever, as they may alter the ECG and trigger ventricular arrhythmias, and, in some cases, receive pharmacological therapy, and undergo implantation of an implantable cardioverter defibrillator (ICD). BrS is a chronic disorder that have no definitive cure to date. Owing to the genetic nature of this condition, family members may also be affected.

The negative impact of several chronic diseases and their therapies on quality of life (QOL) and psychological status has been established in patients with atrial fibrillation (AF), hypertrophic cardiomyopathy (HCM), cancer (2), and an implanted ICD. A few studies have found that patients with hereditable heart conditions, such as catecholaminergic polymorphic ventricular tachycardia (CPVT) (3) and long QT syndrome (LQTS) are psychologically vulnerable and that patients with LQTS and HCM have a higher incidence of heart-focused anxiety (4–7), but data on patients with BrS are limited. A recent retrospective register-based study showed that 15.7% of BrS patients developed new-onset depression or anxiety after diagnosis, especially among symptomatic patients, and that near 75% developed depression or anxiety before death (8); another study showed that BrS patients presented higher mental distress and higher prevalence of type D personality (9). Our study aimed to assess the repercussions of the diagnosis of BrS on QOL and psychological status, not only among patients but also among their relatives, and establish the predictors of these outcomes.

## Materials and methods

### Patient population

This single-centre, prospective, observational study investigated two cohorts: patients diagnosed with BrS and their relatives. The study was approved by the ATS Sardinia Ethics Committee (protocol number 153/2019/CE of 14/05/2019) and conducted in accordance with the principles of the Declaration of Helsinki. All participants provided written informed consent prior to enrolment.

### Inclusion criteria

The inclusion criteria were as follows: (a) age of >18 years; (b) diagnosis of BrS; or (c) relatives. BrS was diagnosed in accordance with the 2015 European Society of Cardiology (ESC) guidelines (1), when patients presented a coved ST-segment elevation ≥2 mm in one or more leads among the right precordial leads V1 and/or V2 positioned in the second, third, or fourth intercostal space, occurring either spontaneously or after provocative drug test with ajmaline. All baseline clinical and ECG data were collected at the time of enrolment.

### Exclusion criteria

The exclusion criteria were as follows: (a) documented diagnosis of another chronic or comorbid condition, including COVID-19; (b) documented history of psychiatric illness or anxiety/depression disorder; and/or (c) cognitive dysfunction that could affect the psychological status.

A control group of healthy individuals matched to the study group by age, sex, and educational level was also enrolled.

### Cardiological evaluation

Cardiological evaluation was performed at diagnosis and every 12 months at our outpatient clinic. After diagnosis, and during each follow-up visit, BrS patients' risk was stratified using the current accepted risk markers: asymptomatic patients with type 1 ECG pattern only induced after ajmaline test were considered at low risk of arrhythmic events (0.3%/year); asymptomatic patients with spontaneous type 1 ECG pattern were considered at intermediate risk (0.8%/year); and symptomatic patients (arrhythmic syncope, nocturnal agonal respiration, aborted SCD) were considered at high risk (1.9%-7.7%/year) and implanted with an ICD (10). Patients underwent additional follow-up visits in the event of symptoms.

### Psychological evaluation

Psychological evaluation was conducted by a psychologist in accordance with the principles indicated by the American Psychological Association (11) through an in-person interview or a telephone call during the COVID-19 pandemic lockdown. The evaluation comprised a sociodemographic form, and questions regarding the perceived level of social support from family and friends.

Six self-report instruments were used to evaluate the different aspects of QOL and psychological status.

• The 12-Item Short Form Health Survey (SF-12) measures the physical and mental aspects of QOL based on two indices: 12-Item Physical Component Summary (PCS-12, from now on “physical QOL score”) and 12-Item Mental Component Summary (MCS-12, from now on “mental QOL score”) scores. Higher scores indicate a better QOL. The normative reference values used for the global, male, and female populations were those of the Italian general population (IGP) (12).
• The Beck Anxiety Inventory (BAI, from now on “anxiety score”) assesses general anxiety levels. Scores between 0 and 7 points are considered normal; scores between 8 and 15 points suggest mild anxiety; and scores higher than 16 points indicate moderate-to-severe anxiety. A cutoff score of 13 points indicates the presence of anxiety symptoms (13).
• The Beck Depression Inventory-II (BDI-II, from now on “depression score”) evaluates the presence and intensity of depression, which starts with a cutoff score of 12 points (14).
• The Impact of Event Scale-Revised (IES-R, from now on “trauma score”) begins with an open-ended question regarding the occurrence of a traumatic event during an individual's lifetime. Individuals who report a traumatic event are asked to answer a 22-item questionnaire that assesses subjective distress related to the aforementioned event. Items are rated on a 5-point scale ranging from 0 (“not at all”) to 4 points (“extremely”). The IES-R comprises three subscales: intrusion, avoidance, and hyperarousal. Scores over 24 points indicate the presence of post-traumatic stress symptoms, while scores over 33 points indicate probable post-traumatic stress disorder (PTSD) (15). In this study, the participants were divided into two groups: those who reported at least one traumatic event and those who did not.
• The Resilience Scale for Adults (RSA, from now on “resilience score”) assesses individual, family, and social resilience protective factors using six subscales: perception of the self, planned future, structured style, social competence, family cohesion, and social resources. The total RSA score is the mean score in these subscales. Higher scores correspond to a greater presence of resilience resources. The reference values used herein were those for the IGP (16).
• The 10-Item Perceived Stress Scale (PSS-10, from now on “stress score”) measures the degree to which different aspects of an individual's life are perceived as uncontrollable, unpredictable, and overloaded, without diagnostic purposes (17). High scores indicate increased stress. The reference values used were those indicated by the authors, with different cutoff scores for men and women.

Table 1 summarises the different instruments used for the psychological evaluation.

**Table 1:** Self-report instruments used to evaluate quality of life and psychological status.

Test		Reference scores	Values
SF-12	*PCS-12*	Level of physical QOL (0–100)	
		Normal (global)	>48.6
		Normal (male)	>49.6
		Normal (female)	>47.5
	*MCS-12*	Level of mental QOL (0–100)	
		Normal (global)	>49.9
		Normal (male)	>48.8
		Normal (female)	>45.2
BAI		Degree of general anxiety (0–63)	
		Minimal	0–7
		Mild	8–15
		Moderate	16–25
		Severe	26–63
BDI-II		Degree of depression (0–63)	
		Minimal	0–13
		Mild	14–19
		Moderate	20–28
		Severe	29–63
IES-R		Degree of post-traumatic stress (0–88)	
		No post-traumatic stress	0–23
		Post-traumatic stress symptoms	≥24
		Probable PTSD	≥33
RSA		Level of global resilience	
		Low	<3.57
		Normal	3.58–4.19
		High	>4.19
PSS-10		Level of stress (0–40)	
		High (male)	≥15.52
		High (female)	≥16.14

BAI, Beck anxiety inventory; BDI-II, Beck depression inventory-II; IES-R, impact of event scale-revised; MCS-12, mental component summary; PCS-12, physical component summary; PSS-10: perceived stress scale; PTSD; post-traumatic stress disorder; QOL, quality of life; RSA, resilience scale for adults; SF-12, SF-12 health survey.

The study population was divided into three groups according to the time of psychological evaluation: group A (T0): 1 month after diagnosis; group B (T1): 1 year after diagnosis; and group C (T2): 2 years after diagnosis.

### Statistical analysis

Data were expressed as percentages, means ± SDs, or medians, as appropriate. The *t*-test was used to compare continuous data and the Wilcoxon signed-rank test and Mann–Whitney *U*-test to analyse non-normally distributed data. Categorical variables were expressed as total numbers (percentages) and compared between groups using the chi-square or Fisher's test, as appropriate. Sociodemographic and clinical variables selected from the univariate analysis (*p* ≤ 0.10) and those considered important for the analysis were entered into multivariate logistic regression to examine their association with QOL (physical and mental QOL scores) and psychological status (anxiety, depression, trauma and resilience scores). Statistical significance was set at *p* < 0.05. Statistical analysis was performed using SPSS version 23 (SPSS Inc., Chicago, IL, USA).

## Results

### Baseline characteristics

Between August 2020 and April 2021, 104 participants were enrolled in the study group. Four patients were excluded owing to withdrawal of consent. The study group finally included 100 individuals: 61 patients diagnosed with BrS and 39 relatives. The control group comprised 105 healthy participants.

The mean age of the patients at diagnosis and psychological evaluation was 49 ± 12 and 51 ± 12 years, respectively. Forty-three (70.5%) were probands; 20 (32.8%) had a family history of SCD; and 27 (44.3%) had blood relatives with the same diagnosis.

The mean age of the relatives was 47 ± 12 years. Twenty-two (56.4%) were blood relatives, of whom nine (23%) had not yet undergone family screening for BrS, and whether they were affected at the time of the psychological evaluation was unknown. The remaining 44% were partners. The baseline characteristics of the study population are summarised in Table 2.

**Table 2:** Baseline characteristics of the study population.

		Total sample (*n* = 100)	Patients (*n* = 61)	Family members (*n* = 39)	Control group (*n* = 105)	*p*
Male		46 (46%)	35 (57.4%)	11 (28.2%)	47 (45%)	0.117 NS
Age (years)		49 ± 12	51 ± 12	47 ± 12	49 ± 14	0.380 NS
Marital status	Single	27 (27%)	17 (27.9%)	10 (25.6%)	28 (26.7%)	0.601 NS
	Married/cohabitant	59 (59%)	35 (57.4%)	24 (61.5%)	61 (57.1%)	
	Separated/divorced	12 (12%)	7 (11.5%)	5 (12.8%)	8 (7.6%)	
	Widow/widower	2 (2%)	2 (3.3%)	0	8 (7.6%)	
Employment		82 (78%)	50 (78%)	32 (78%)	83 (79%)	0.650 NS
Education status	Midle school	33 (33%)	22 (36%)	11 (28%)	33 (31.5%)	0.924 NS
	High school	6 (6%)	3 (5%)	3 (8%)	7 (7%)	
	Associate degree	32 (32%)	17 (28%)	15 (38.5%)	35 (33%)	
	Bachelor degree	26 (26%)	17 (28%)	9 (23%)	27 (25.5%)	
	Post graduate	3 (3%)	2 (3%)	1 (2.5%)	3 (3%)	
Subgroup	Group A (T0)		20 (32.8%)	16 (41%)		
	Group B (T1)		24 (39.3%)	15 (38.5%)		
	Group C (T2)		17 (27.9%)	8 (20.5%)		
Proband			43 (70.5%)			
ICD			5 (8.2%)			
Arrhythmic risk	Low		48 (78.7%)			
	Intermediate		7 (11.5%)			
	High		6 (9.8%)			
Family history of SCD			20 (32.8%)	0		
Other patients in the family			27 (44.3%)	100%		
Family members	Partners			17 (43.6%)		
	Blood relatives			22 (56.4%)		

*p*, patients vs. control group.

ICD, implantable cardioverter-defibrillator; NS, non-significant; SCD, sudden cardiac death.

### Group A

Twenty patients and sixteen relatives (*n* = 36) were evaluated at a mean period of 10 ± 22 days after diagnosis.

**Figure 1:**
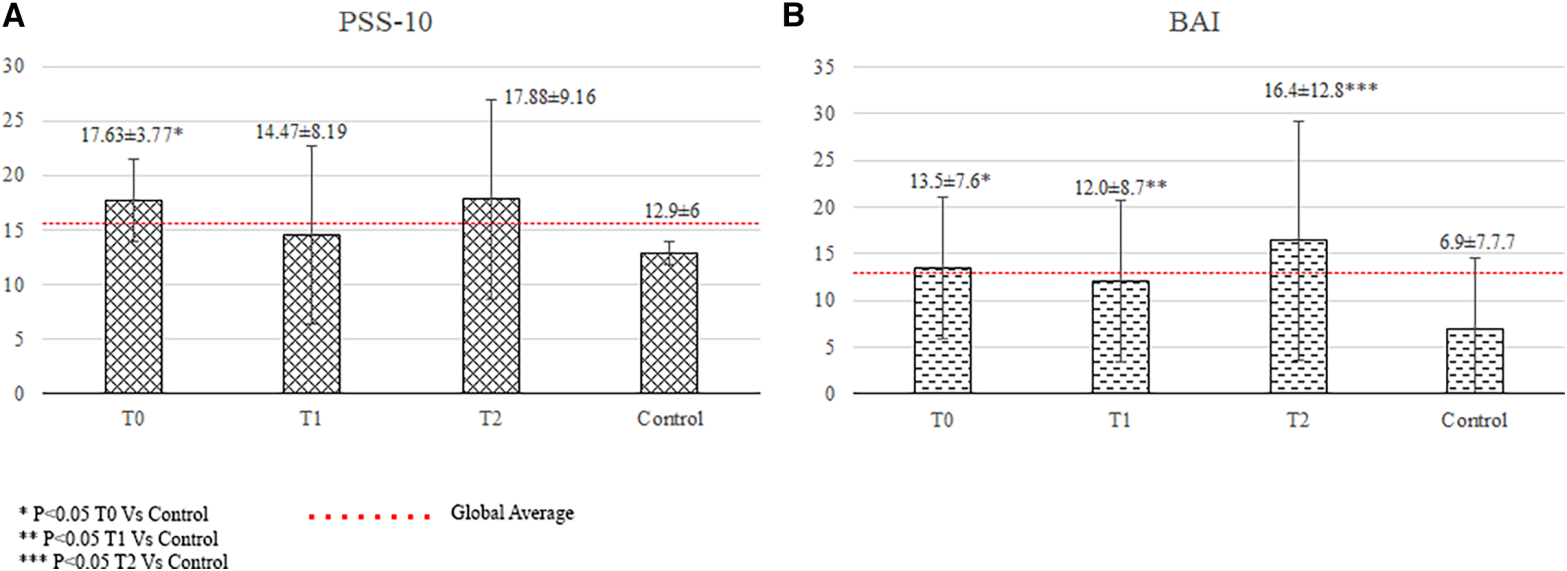
Quality of life and psychological status of patients. BAI, Beck Anxiety Inventory (anxiety score); MCS-12, mental component summary of the SF-12 health survey; PCS-12, physical component summary of the SF-12 health survey; RSA, resilience scale for adults (resilience score); T, time of the psychological evaluation; T0, one month after diagnosis; T1, one year after the diagnosis; T2, two years after the diagnosis.

The patients showed significantly better physical QOL scores (54.1 ± 6.5 vs. 50.1 ± 8.0, *p* = 0.014), worse mental QOL scores (43.2 ± 11.8 vs. 49.6 ± 9.1, *p* = 0.018) and significantly higher anxiety scores than did the controls (9.9 ± 6.6 vs. 6.9 ± 7.7, *p* = 0.024; Figure 1). The patients had a significantly higher incidence of a traumatic event at any moment during their lifetime than the controls (45% vs. 19%, *p* = 0.019; Figure 1).

The relatives showed significantly higher anxiety (13.5 ± 7.6 vs. 6.9 ± 7.7, *p* < 0.001), and stress scores (17.63 ± 3.77 vs. 12.90 ± 6.0, *p* = 0.02) than did the controls. Their depression scores were lower than the controls' (7.2 ± 3.6 vs. 5.7 ± 6.4, *p* = 0.028), but values were within normal range.

### Group B

**Figure 2:**
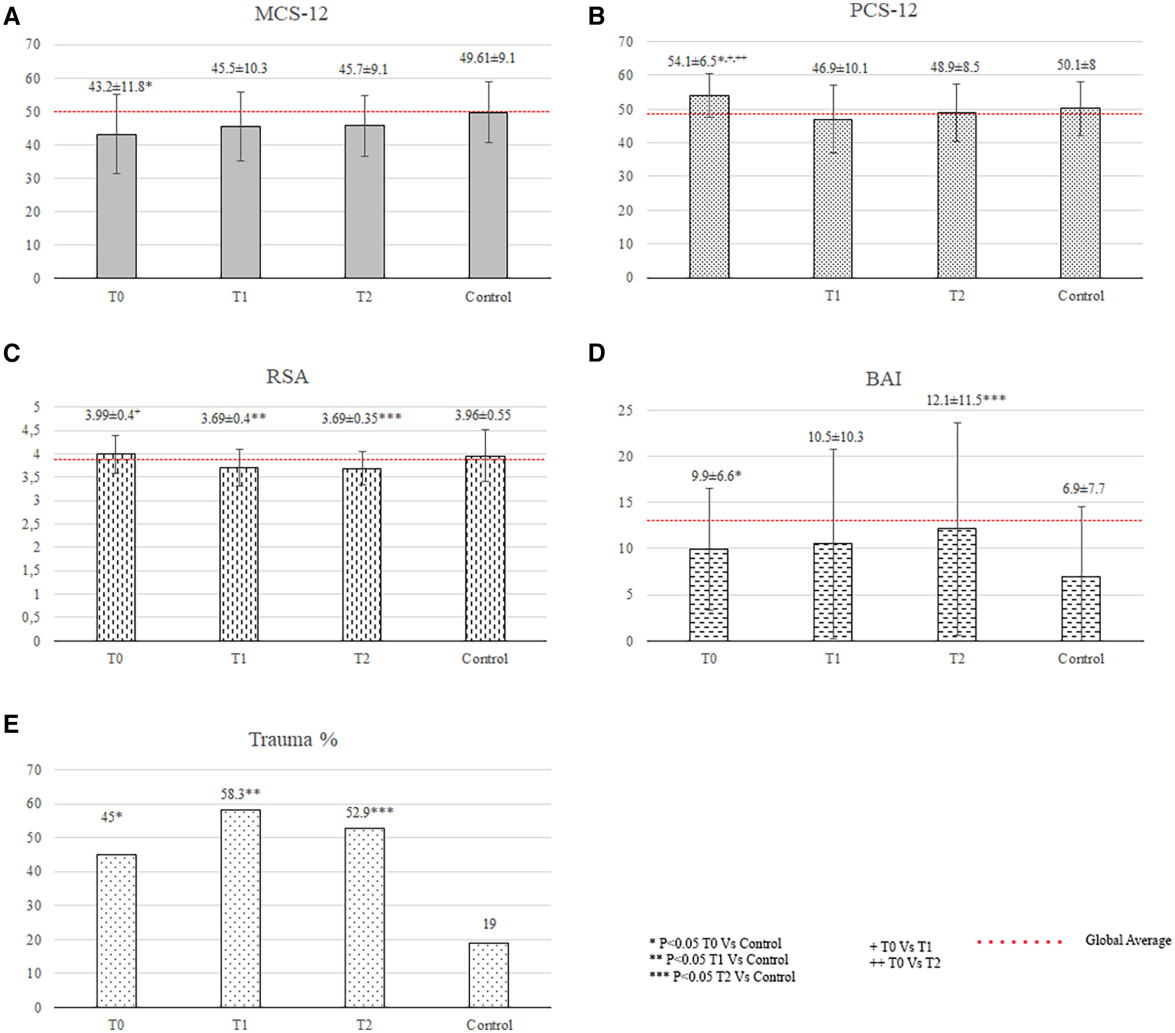
Quality of life and psychological status of family members. BAI, Beck anxiety inventory; PSS-10, 10-item perceived stress scale; T, time of the psychological evaluation; T0, one month after diagnosis; T1, one year after the diagnosis; T2, two years after the diagnosis.

Twenty-four patients and fifteen relatives (*n* = 39) underwent psychological evaluation at a mean time from diagnosis to evaluation of 12 ± 3 months. The patients had a lower total resilience score (3.69 ± 0.40 vs. 3.96 ± 0.55, *p* = 0.008) and a significantly higher incidence of traumatic events than the controls (58.3% vs. 19%, *p* < 0.001; Figure 2).

The relatives showed significantly higher anxiety scores (12.0 ± 8.7 vs. 6.9 ± 7.7, *p* = 0.005; Figure 2) than did the controls.

### Group C

Seventeen patients and eight relatives (*n* = 25) underwent psychological evaluation at a mean period of 22 ± 5 months after diagnosis.

The patients had significantly higher anxiety levels (anxiety score: 12.1 ± 11.5 vs. 6.9 ± 7.7, *p* = 0.032), lower total resilience scores (3.69 ± 0.35 vs. 3.96 ± 0.55, *p* = 0.019), and higher incidence of reported traumatic events (52.9% vs. 19%, *p* = 0.005) than the controls (Figure 1).The relatives showed significantly higher anxiety scores than did the controls (16.4 ± 12.8 vs. 6.9 ± 7.7, *p* = 0.006), which were above the cutoff score for anxiety disorders (Figure 2).

Detailed data from the psychological tests are presented in Table 3, while detailed data on traumatic events among the patients and their relatives are provided in Supplementary Table S1.

**Table 3:** Quality of life and psychological status in patients and family members after diagnosis of cardiac Brugada syndrome with control group.

QoL and psychological variables	Group 1				Group 2				Group 3				Healthy Controls
	Pt *n* = 20	*p*	FM *n* = 16	*p*	Pt *n* = 24	*p*	FM *n* = 15	*p*	Pt *n* = 17	*p*	FM *n* = 8	*p*	*n* = 105
PCS-12 (physical QOL)	54.1 ± 6.5*	0.014	48.5 ± 9.7	NS	46.9 ± 10.1	NS	52.4 ± 7	NS	48.9 ± 8.5	NS	47.8 ± 8.8	NS	50.1 ± 8.0
MCS-12 (mental QOL)	43.2 ± 11.8*	0.018	47.3 ± 8.6	NS	45.5 ± 10.3	NS	45.2 ± 12.3	NS	45.7 ± 9.1	NS	45.9 ± 10.3	NS	49.6 ± 9.1
BAI (Anxiety)	9.9 ± 6.6*	0.024	13.5 ± 7.6*	<0.001	10.5 ± 10.3	NS	12.0 ± 8.7*	0.005	12.1 ± 11.5*	0.032	16.4 ± 12.8*	0.006	6.9 ± 7.7
BDI-II (Depression)	8.2 ± 7.0	NS	7.2 ± 3.6*	0.028	5.3 ± 4.5	NS	5.3 ± 5.0	NS	5.5 ± 5.5	NS	7.9 ± 7.9	NS	5.7 ± 6.4
IES-R (%Subjects who have reported a traumatic event)	9 (45%)*	0.019	6 (37.5%)	NS	14 (58.3%)*	<0.001	6 (40%)	NS	9 (52.9%)*	0.005	3 (37.5%)	NS	20 (19%)
IES-R total score (Trauma)	30.8 ± 23.5	NS	35.2 ± 22.3	NS	25.1 ± 23.6	NS	33.0 ± 26.3	NS	29.5 ± 17.0	NS	24 ± 31.3	NS	31.6 ± 23.3
Pss-10 score (Stress)	14.55 ± 6.02	NS	17.63 ± 3.77*	0.02	15.04 ± 6.77	NS	14.47 ± 8.19	NS	15.24 ± 7.05	NS	17.88 ± 9.16	NS	12.90 ± 6.0
RSA score (Resilience)	3.99 ± 0.40	NS	4.05 ± 0.29	NS	3.69 ± 0.40*	0.008	3.95 ± 0.47	NS	3.69 ± 0.35*	0.019	3.75 ± 0.28	NS	3.96 ± 0.55

Values are mean ± SD.

Pt, patients; FM, family members; PCS-12, physical component summary of the SF-12 health survey (SF-12), MCS-12 mental component summary of the SF-12 health survey (SF-12); BAI, Beck anxiety inventory; BDI-II, Beck depression inventory-II; IES-R, the impact of event scale-revised; PSS-10, perceived stress scale-10; RSA, resilience scale for adults.

* *P* < 0.05 compared with control group.

### Variables associated with QOL and psychological status

The multivariate logistic regression model identified female sex as the only independent variable significantly associated with worse mental QOL scores among the patients at T0 (odds ratio = 0.10; 95% confidence interval = 0.05–0.94; *p* = 0.04). No other variables, including high arrhythmic risk or being implanted with an ICD, were associated with QOL or psychological status among the patients at T1 and T2 or among the relatives at T0, T1, or T2. There were no difference in QOL and psychological status between patients with spontaneous and ajmaline-induced type 1 ECG pattern. A detailed description of the logistic regression analysis results is provided in Table 4.

**Table 4:** Univariate and multivariate analysis of risk factors associated with MSC-12 at T0.

*MCS-12* at T0	Univariate		Multivariable	
	OR (95% CI)	*P*	OR (95%CI)	*P*
Gender (♀)	6 (0.81–44.35)	0.07	0.10 (0.05–0.94)	0.04
Age at diagnosis (≥48yo)	0.5 (0.08–3.17)	0.45	6.2 (0.44–86.82)	0.17
Family support	6 (0.81–44.35)	0.07	0.08 (0.03–2.95)	0.14
Arrhythmic risk	1.4 (0.10–18.61)	0.79	0.23 (0.10–5.81)	0.37

MSC-12, mental component summary of the SF-12 health survey [SF-12].

## Discussion

The main findings of the study were as follows: (1) the diagnosis of BrS had a negative impact on QOL and psychological status in both patients and their relatives; (2) the main psychological response to the diagnosis of BrS among patients and relatives was anxiety; (3) patients also showed abnormal QOL and reduced resilience, while relatives also reported stress; (4) the psychological impact started at diagnosis and persisted until 2 years of follow-up; and (5) female sex was independently associated with low mental QOL scores at T0 in patients.

### QOL

The patients showed significantly better physical QOL scores than did the controls at T0, consistent with previous findings on patients with LQTS (5). A combination of denial and absence of symptoms in the majority of our patients may account for this result. The first phase of disease adjustment is characterised by shock, denial, and disbelief (18), which are defensive reactions mobilised to relieve anxiety and other threatening emotions elicited by the diagnosis and are useful in providing time to process the distressing information more gradually. Denial can minimise alarming news received and even completely reject the severity and future implications (19). Good physical-related QOL has been associated with asymptomatic status in HCM and AF (20, 21). The fact that BrS does not affect physical performance, and, as in our series, many patients were asymptomatic (22), may account for their higher physical QOL.

The patients showed a significant reduction in the mental QOL score at T0, also consistent with prior data on patients with LQTS (23). Although the cause of a reduced mental QOL in LQTS is still unknown, prior research (4) has indicated that emotional health is harmed due to the inherent uncertainty surrounding all elements of diagnosis, symptoms, and therapy, which may also be present in the case of BrS patients. In addition, a recent study has shown that individuals with a suspected BrS diagnosis had significantly higher mental distress than already diagnosed BrS patients (9), supporting the negative influence of uncertainty on the psychological status. The waiting period between the initial suspicion and definitive diagnosis may also play a role, as patients are exposed to alarming information from non-medical sources. Further studies will be needed to establish the impact of the waiting period from suspicion to diagnosis on mental QOL in these patients.

The absence of symptoms during follow-up and continued surveillance in a specialised tertiary referral clinic may account for the normalisation of QOL in our patients at follow-up (T1 and T2).

The relatives did not show changes in QOL at any evaluation period.

### Psychological status

The patients presented with mild levels of anxiety at all evaluation periods and had significantly higher anxiety scores than the controls at T0 and T2. A strong association between heart disease and anxiety is known in the literature (24). The diagnosis of a chronic illness disrupts a person's life and requires ongoing psychological adjustment (19), remaining a potential source of anxiety throughout the lifespan (25). A study that investigated patients with LQTS from 1 to 10 years after diagnosis reported daily difficulties and limitations over the long term, which undermined their sense of control over the disease, generating anxiety (26). The reduction in resilience resources observed at T1 and T2 might have played a role in the development of long-term anxiety in the patients.

Resilience is the ability to successfully maintain or recover mental health during adversities using protective resources within oneself and one's family and social environment. It has been associated with good adaptation to illness (27). Chronic illnesses are undeniable stressors (19), and their negative impact on resilience has been previously demonstrated (28). In our cohort, the patients evaluated at T0 showed normal resilience, whereas those evaluated at T1 and T2 reported significantly lower resilence scores; these findings indicate that their usual cognitive, affective, behavioural, and social coping responses (28) have become ineffective, underlining the need to implement multidimensional interventions that promote the development and enhancement of personal and family resilience during follow-up.

The trauma scores were not significantly different between the patients and controls, as previously observed in patients with CPVT (3). A life-threatening illness is not necessarily considered a traumatic event but may present with traumatic symptoms and PTSD. In a study on patients with cancer, myocardial infarction, and stroke (29), the most powerful prognostic factor for PTSD was the degree of life threat subjectively experienced at the onset of an acute medical event, such as symptom occurrence. The absence of symptoms in 96% of our cohort may account for the normal levels of post-traumatic stress. However, the number of patients who reported having experienced trauma during their lifetime was significantly higher than that of controls at all evaluation periods. Some of these events were related to the diagnosis of BrS or its possible consequences, such as a family history of SCD and facts about their own or a relative's disease or diagnostic process. This provides interesting information about how they experienced these events and their difficulties in coping with them (Supplementary Table S1).

The predominant affective response among family members after the diagnosis of BrS was anxiety, which was mild at T0 and T1 and reached levels of clinical concern at T2. Anxiety in partners (4) and parents (30, 31) of patients with LQTS has been previously documented; they reported experiencing fear of death, perpetual uncertainty and discomfort in interpreting the signs and symptoms of the disease, and continuous hypervigilance combined with frustration owing to a lack of knowledge of the disease from health professionals (26). Our study showed that this phenomenon is also present in patients with BrS, emphasising the impact that this type of diagnosis can have on the entire family system and the emotional balance of partners and the need to offer psychological support not only to patients but also to the family, starting immediately after the diagnosis.

Our data showed that the relatives presented significantly higher stress scores at T0 than did the controls, proving that the diagnosis of a loved one qualifies as an acute stressor for family members. Further studies are needed to better characterise the stress response among the family members of patients with BrS.

Depression scores were normal at all evaluation times in BrS patients and their relatives. Family members reported significantly higher depression scores than controls at diagnosis, which nevertheless fell within the normal range, consistent with other studies in relatives of LQTS patients (4).

Female sex was independently associated with reduced mental QOL scores among the patients at the time of diagnosis, which is consistent with the findings of previous studies that focused on patients diagnosed with LQTS and HCM (5). The mechanism underlying the increased susceptibility in women remains unclear, but it has been suggested that may be a consequence of living with ill-related uncertainty, which seems to have a different impact on men and women's health.

### Clinical implications

In order to promote the psychological well-being of patients with BrS and their families, given the results presented here and the most recent studies (8, 9), it appears necessary to implement in routine clinical activities a psychological protocol, including an assessment phase, low-complexity, and, when necessary, high-complexity interventions (32).

The initial assessment identifies, at the time of diagnosis, psychological problems, needs, and resources of the person in order to construct an individualized intervention. Low-complexity interventions include psycho-education and psychological counseling. Psychologists, in collaboration with the multidisciplinary team, carry out the psycho-education, promoting understanding and management of the disease, adherence to treatment, and reintegration into normal daily life. Psychological counseling aims to accompany the person through his or her specific difficulties, such as acceptance of the diagnosis, moments of crisis, motivation for change, and recognition and management of anxiety. In particular, learning psycho-body relaxation, breathing and mindfulness techniques can help the person develop awareness of his or her mental processes and manage anxiety effectively. If more structured and resistant psychological issues exist, like depression, panic disorder, obsessive-compulsive disorder or insomnia, it is necessary to implement an individual or couples psychotherapy.

The psychological impact of the BrS diagnosis is undeniable, and must grant psychological support, but it is important to emphasize that current guidelines and experts recommend reevaluating the diagnosis of many patients, and that candidates for ajmaline test should be carefully chosen (33, 34). It is also essential to explain clearly the potential drawbacks of the test. Numerous studies have demonstrated the high sensitivity and moderate specificity of ajmaline test, leading to a significant rate of false-positive results (35). The diagnosis of BrS is now only considered probable in patients with a drug-induced type 1 ECG pattern and no other clinical, family, or genetic characteristics; their arrhythmic risk is very low (10). A decrease in false-positive diagnoses would shield people at very low risk of SCD from many of the detrimental psychological effects of a potential diagnosis, as Six et al. (9) have shown that the prospect of receiving a BrS diagnosis carries greater psychological weight, even for those negative to the diagnostic test, than a prior BrS diagnosis.

### Limitations

Due to limitations resulting from the COVID-19 pandemic, each group was assessed only once (at T0, T1, and T2). As a result, it was not possible to determine intra-individual differences in QOL and psychological status. This also resulted in low number of individuals in each group, limiting the statistical power of the results.

The small sample size, particularly of the high-risk group, could be the reason for the lack of correlation shown between the psychological and QOL outcomes with high arrhythmic risk status.

Our study did not evaluate QOL and psychological status pre-diagnosis, nor the impact of the suspected diagnosis in individuals in which the diagnosis was excluded.

To reduce the individual influence of the COVID-19 on the results of the QOL and psychological evaluation, having contracted the disease was an exclusion criterion for enrolment. However, it is possible that part of the negative results in terms of QOL, anxiety, depression, stress, and resilience are a consequence of the global COVID-19 pandemic and the stress of social isolation.

Conducting a new prospective study evaluating a similar group of patients and relatives before diagnosis, and at T0, T1 and T2, including also those individuals in which BrS was excluded, and increasing the sample size would solve these limitations and clarify the pandemic's influence on the current results.

## Conclusions

The diagnosis of BrS has a negative impact on different areas of the psychological sphere in patients (QOL, resilience, and anxiety) and their relatives (anxiety and stress) that starts at diagnosis and persists for 2 years. Female sex is independently associated with a worse mental-related QOL in patients at diagnosis. Psychological interventions should be available to patients and their families at diagnosis and during follow-up.

## Data Availability

The data that support the findings of this study are available from the corresponding author upon reasonable request.
