# Supplementary material for: Diagnosis of Brugada syndrome affects quality of life and psychological status
Source: Front Cardiovasc Med. 2024 Jul 3;11:1429814. doi: 10.3389/fcvm.2024.1429814 (PMC11251888; doi:10.3389/fcvm.2024.1429814)
Supplement: Supplementary file 1 [file Table1.docx]

Table 1 (Supplementary Material): Nature of trauma. Abbreviations: BrS, Brugada syndrome.

|  | Group 1 | | Group 2 | | Group 3 | | Healthy Controls |
| --- | --- | --- | --- | --- | --- | --- | --- |
| Nature of trauma | Patients n=20 | Family  Members n=16 | Patients n=24 | Family  Members n=15 | Patients n=17 | Family  Members n=8 | n=105 |
| No trauma | 9 (45%) | 0 | 7 (29,2% ) | 0 | 5 (23,4%) | 0 | 85 (81%) |
| Own diagnosis of BrS | 3 (15%) | 0 | 0 | 0 | 3 (17,6%) | 0 | 0 |
| SCD in the family | 8 (40%) |  | 6 (25%) | 2 (13%) | 6 (35,3%) |  | 0 |
| Diagnosis of BrS in a family member | 6 (30%) | 16 (100%) | 1 2(50%) | 15(100%) | 9(52,9%) | 8 (100%) | 0 |
| Diagnosis of other life-threatening disease in a family member | 2 (10%) | 1 (6,3%) | 3 (12,5%) | 1 (6,7%) | 0 | 0 | 4 (4%) |
| Serious accident | 1 (5%) | 0 | 0 | 1 (6,7%) | 1 (5,9%) | 1 (12,5%) | 6 (6%) |
| Sudden grief | 1 (5%) | 2 (12,5%) | 5 (20,8%) | 0 | 1 (5,9%) | 1 (12,5%) | 4 (4%) |
| Suicide/homicide | 0 | 1 (6,3%) | 0 | 1 (6,7%) | 1 (5,9%) | 0 | 1 (1%) |
| Aggression | 0 | 0 | 0 | 0 | 1 (5,9%) | 0 | 2 (2%) |
| Catastrophe | 0 | 0 | 0 | 0 | 0 | 0 | 1 (1%) |
| Another trauma | 2 (10%) | 0 | 1 (4,2%) | 0 | 0 | 0 | 2 (2%) |
